# Detection of Arsenic Acid

**Published:** 1842-04

**Authors:** 


*Detection of Arsenic Acid*. By M. Elsner.—It is well known that M. Runger discovers free sulphuric acid by covering a porcelain dish with a solution of one part of sugar and thirty parts of water, heating the dish by exposure to steam till it acquires the same heat, and then dropping on it the liquid supposed to contain the free sulphuric acid. A black color indicates the presence of this acid, because the greater number of other free acids do not decompose the sugar in this manner.

I have found that arsenic acid acts in a peculiar manner, producing on the porcelain coated with sugar a beautiful scarlet red color. The reaction is sensible with a liquid containing only $\frac{1}{1200}$ of arsenic acid. The action of the arsenic acid produces on the sugar ulmic acid, which brings the former acid to an inferior degree of oxydation.—*Journal of Franklin Institute*, from *Annal des Mines*.

